# United States Salary List and the Civil Service Law, Rules and Regulations, with Specimen Examination Questions in the Custom House, Post Office, and Classified Departmental Service

**Published:** 1883-11

**Authors:** 


**United States Salary List and the Civil Service Law, Rules and Regulations, with Specimen Examination Questions in the Custom House, Post Office, and Classified Departmental Service.** HENRY N. COPP, Attorney and Counselor-at-Law, Washington, D. C. 1883.

The 160 pages of this book give a good deal of solid information. All the government salaries are given, from the President's $50,000 to the postmaster with $500. The specimen examination papers presented to candidates for admittance to the different departments of the civil service are interesting. We venture to say that three-fourths of the medical students at our many diploma mills could not answer the questions given to the candidate for the position of letter carrier. The book is a useful one for all young men and women who have not decided upon a calling in life. The government offers lucrative and honorable employment; and under the rules inaugurated by the civil service reform movement, the best qualified candidates are supposed to have the preference in all appointments.

